# The development of interoceptive hunger signals

**DOI:** 10.1002/dev.22374

**Published:** 2023-02-07

**Authors:** Richard J. Stevenson, Johanna Bartlett, Madeline Wright, Alannah Hughes, Brayson J. Hill, Supreet Saluja, Heather M. Francis

**Affiliations:** ^1^ Department of Psychology Macquarie University Sydney New South Wales Australia

**Keywords:** associative learning, homeostatic model, hunger, internal state, interoception

## Abstract

Hunger is often reported when people experience certain internal sensations (e.g., fatigue) or when they anticipate that a food will be good to eat. The latter results from associative learning, while the former was thought to signal an energy deficit. However, energy‐deficit models of hunger are not well supported, so if interoceptive hungers are not “fuel gauges,” what are they? We examined an alternate perspective, where internal states signaling hunger, which are quite diverse, are learned during childhood. A basic prediction from this idea is offspring–caregiver similarity, which should be evident if caregivers teach their child the meaning of internal hunger cues. We tested 111 university student offspring–primary caregiver pairs, by having them complete a survey about their internal hunger states, alongside other information that may moderate this relationship (i.e., gender, body mass index, eating attitudes, and beliefs about hunger). We observed substantial similarity between offspring–caregiver pairs (Cohen's *d*s from 0.33 to 1.55), with the main moderator being beliefs about an energy‐needs model of hunger, which tended to increase similarity. We discuss whether these findings may also reflect heritable influences, the form that any learning might take, and the implications for child feeding practices.

## 1 INTRODUCTION

People report being hungry when they experience certain internal sensations (e.g., rumbling stomach) or when they anticipate that a food will be good to eat (e.g., Cofer & Appley, 1964; Reber, 1985). This division between internal sensations and mental anticipation maps onto a “two‐process” view of hunger (e.g., Cannon & Washburn, 1912; May et al., 2012; Weingarten, 1985). One process (“appetite”) arises from learning associations between food cues and their sensory (i.e., hedonic) and postingestive consequences (e.g., see chocolate, feel appetite for chocolate; Espel‐Huynh et al., 2018; May et al., 2012; Papies et al., 2020). The other process has been presumed to have a homeostatic origin, arising from changes in bodily fuel needs, with this being signaled via certain internal sensations (Cannon & Washburn, 1912; Monello & Mayer, 1967; Schacter, 1968). While support for learned models of hunger is robust (Espel‐Huynh et al., 2018; May et al., 2012; Papies et al., 2020), there is now considerable skepticism over the viability of homeostatic models (e.g., Rogers & Brunstrom, 2016; Stricker, 1984; Strubbe & Woods, 2004; Woods et al., 2000). This creates a problem. If internal sensations of hunger are not “fuel gauges” indexing energy depletion, the question arises as to what they are. It is this issue that we address here by examining their developmental origins.

Internal signals of hunger (e.g., rumbling stomach, feeling cold, fatigue, irritability) have been presumed to represent a largely “innate” system present since birth, which directs the organism to seek food (see Harshaw, 2008 for a historical perspective). Such internal signals were thought to reflect fuel status, be it blood glucose (e.g., Campfield et al., 1996; Mayer, 1953; Melanson et al., 1999), circulating lipids (Kennedy, 1953), or some other physiological marker (Kissileff & Van Itallie, 1982). When the requisite physiological marker changes (e.g., a decline in blood sugar), this causes the onset of an internal hunger signal, thereby signaling the need to eat. This type of homeostatic view can be readily appreciated by looking at thirst. Here, a dry mouth—the internal signal—reliably indicates fluid deficit, and when this deficit is corrected (i.e., a return to homeostasis) the sensation dissipates (Brunstrom, 2002; Labbe et al., 2009; Rolls et al., 1980). While this homeostatic view of thirst is well supported (and noting that there is also learned anticipatory drinking), a homeostatic view of hunger is not. The main reason is that the human body does not readily run out of fuel, at least over a matter of several hours, days, or even weeks (e.g., Rogers & Brunstrom, 2016; Stricker, 1984; Strubbe & Woods, 2004; Woods et al., 2000). Importantly, this is not to say that physiology is unimportant, rather that it exerts its influence in other ways (e.g., see Davidson et al., 2019; Liu & Kanoski, 2018).

Instead of assuming that internal hunger sensations are “innate,” another perspective has been that their meaning is learned (Bruch, 1969; Hebb, 1949; Richter, 1927). The most recent proponent for this view has been Harshaw (2008). The principal source of human evidence concerns infants (e.g., Hetherington, 2017; Hodges et al., 2013), with the idea that breastfeeding is particularly beneficial as the child can learn to self‐regulate intake, in contrast to parent‐controlled bottle feeding (e.g., Ventura, 2017). However, breast or bottle feeding is not informative about how certain internal states may come to signal hunger. The animal literature has some suggestive findings. Changizi et al. (2002) observed that rat pups cannot respond appropriately to food deprivation until they encounter food and eat in that state. They suggest that internal state serves as an occasion setter, such that cues linked to food come to elicit an appetitive response only in this state (Davidson, 1993; Holland, 1991). Put in human terms, one comes to learn that when I feel X (e.g., a rumbling stomach), food will be good to eat *now*.

One consequence of this type of learning model is that internal signals of hunger should be quite idiosyncratic. This is because many different bodily sensations could potentially be predictive of food being good to eat now. Monello and Mayer (1967) reported that there are “…many and diverse sensations of hunger…” (p. 261), with many not identifying abdominal‐related sensations as hunger. In a further study, Harris and Wardle (1987) concluded: “It proved impossible to identify a specific subset or constellation of hunger symptoms which were characteristically experienced by hungry people” (p. 154). Harris and Wardle (1987) also reported that 40% of their sample did not report abdominal‐related sensations prior to a meal. Other studies have found that many people (perhaps up to 50%) cannot recognize the stomach contractions that occur when this structure is empty (Stunkard & Fox, 1971; Whitehead & Drescher, 1980). The internal sensations that characterize hunger do seem to be idiosyncratic.

There has been little study of how humans learn that certain internal sensations come to signal that food will be good to eat now. It seems reasonable to assume that this will occur during childhood. Indeed, there has been a lot of interest in how eating‐related parenting styles (e.g., Beckers et al., 2021; Blissett et al., 2006; Galloway et al., 2010) can dysregulate appetitive control (i.e., override internal signals of hunger and satiety). Our contention is that parenting is also important for teaching children the meaning of hunger, and that this may occur during the period from weaning onward. Certainly, by the time children attend school, they seem to understand that certain internal states indicate hunger (e.g., Bennett & Blissett, 2014). Once such learning has occurred, it should be resistant to change, as it will be the product of intermittent reinforcement. That is, on each occasion an indicative internal state occurs (e.g., a tummy rumble), it will be followed *only* occasionally by food, but when it is, the food will probably taste good. Consequently, evidence of this learning should persist into adulthood as a pattern of internal signals linked to eating, differing between individuals, but with greater similarity to one's primary caregiver during childhood (i.e., one's “teacher”) than to a stranger. The aim here is to test this idea by seeing if reports of internal hunger sensations are similar between young adults and the person who was their primary caregiver during childhood.

To explore internal hunger sensations, we asked students (“offspring”) and their primary caregiver during childhood to complete a modified version of Monello and Mayer's (1967) Hunger survey, so we could test for response similarity. In addition, we also selected a number of potential moderating variables to explore, with these being collected from both offspring and caregiver. The first were demographic, namely gender, noting that primary caregivers would most likely be mothers (e.g., Dupuy et al., 2021; Galloway et al., 2010), and Body Mass Index (BMI), which might influence the type of hunger sensations that parents teach or that children learn (e.g., Schachter, 1968). Second, we undertook to measure caregiver and offspring beliefs about hunger (see Assanand, Pinel, & Lehman, 1998), creating a new scale for this purpose to assess homeostatic and learning‐related beliefs, again to see if this impacted the type of hunger sensations that are acquired. If one believes that internal sensations index “fuel levels,” then this may lend more weight to teaching this to one's offspring (i.e., nobody wants to “run out of fuel”). Finally, offspring and caregivers completed the Three‐Factor Eating Questionnaire (TFEQ revised form; Cappelleri et al., 2009). The three dimensions of the TFEQ are important as they reflect core areas of appetitive dysfunction—uncontrolled eating (i.e., excessive hunger), restrained eating (i.e., excessive control), and emotional eating (i.e., eating in response to emotion)—all of which might be expected to influence caregiver teaching and offspring learning. We did not predict any pattern of moderation here, as there is no prior work to guide.

## 2 METHOD

### 2.1 Participants

The study was powered to find a medium effect size (Cohen's *d* = 0.6) approximately equivalent to a correlation of .3. This value was selected based upon other studies examining similarities in psychological variables (e.g., attitudes) between primary caregivers and their offspring that may reflect learning (e.g., Degner & Dalege, 2013). To detect a correlation of around .3 with alpha set at .05 and an 80% chance of rejecting the null if H1 were true requires at least 85 caregiver–offspring pairs.

Caregiver–offspring pairs were recruited in the following way. Potential student participants (i.e., who would serve as “offspring” for this study) viewed an electronic advertisement on the first‐year psychology participant pool homepage. The study advertisement advised prospective student participants that if they wished to take part, they would first need to contact and secure the cooperation of the person who primarily cared for them as a child. It was made clear at this stage that their (i.e., the student) survey could not be completed without both entering the contact details for their primary caregiver and having secured initial permission for their caregiver to be approached by the research team.

When students contacted their primary caregiver, they were asked to discuss the following: (1) Were they interested in taking part? (2) Did they meet the eligibility requirements (which also applied to the student offspring)? (3) Did they agree to make their name and contact details known to the research team? What constituted “taking part” was the same for student offspring and their primary caregivers (typically mothers). This involved completing a 30‐min online survey about hunger and doing so around 30 min before eating a main meal. There were two exclusion criteria: first, any disorder/medication that might affect hunger; second, a current or past history of eating disorders. The advert also described how once the student offspring had completed their survey, the research team would then harvest their primary caregiver details from this, and then send their primary caregiver the survey website link. Primary caregivers were then free to either ignore this request (i.e., decline participation) or to complete the survey. Students were awarded course credit for taking part. Primary caregivers were thanked for taking part but did not receive any reimbursement.

One hundred and sixteen caregiver–offspring pairs successfully completed their surveys (noting an additional 69 student offspring also completed the surveys but did not have accompanying parent data, and that all of the student sample [*n* = 185] were included in a further study). Data from five participants were excluded due to either failing all four check questions (see Section 2.3 below) or reporting a current eating disorder, leaving 111 caregiver–offspring pairs for analysis. The offspring group had a mean age of 22.1 years (*SD* = 8.2) and the caregivers had a mean age of 52.2 years (*SD* = 8.7). The primary caregiver was mainly the mother (*n* = 105), with just a few fathers (*n* = 6). Additional sample details are provided in Section 3.

The study protocol was approved by the Macquarie University Human Research Ethics Committee (Project ID 11337) and informed consent was provided by each participant.

### 2.2 Materials

#### 2.2.1 Hunger survey

The Hunger survey was based upon Monello and Mayer's (1967) “Hunger‐Satiety Questionnaire.” Before starting the survey, participants were asked five general questions about their current level of hunger: (1) Time since last meal; (2) Current hunger; (3) Urge to eat; (4) Thoughts about food; and (5) How much food would be eaten now. Question (1) had four response options (In the last hour, In the last 1–2 h, In the last 2–4 h, and More than 4 h ago), while the remainder used 5‐point category scales (*Not at all* [1] to *Extremely* [5]). These questions served to establish whether participants were in the requisite motivational state to complete the survey (i.e., hungry).

The main part of the Hunger survey then commenced, focusing on the different internal states that participants associated with hunger. This part was composed of six blocks of questions, presented in a fixed order. The response format for all questions was the same, using a 6‐point category scale ([1] *Not at all*; [2] *Very weak*; [3] *Weak*; [4] *Moderate*; [5] *Strong*; [6] *Very strong*). This enabled participants to both report the absence or presence of that state, and if present, the intensity of the signal. Block one concerned mood states. Participants were asked “When you are hungry, which of the following moods do you usually experience?” The following were evaluated: Irritable; Nervous and tense; Bored; Cheerful; Excited; Impatient; Calm and relaxed; Content; and Apprehensive. Block two concerned the stomach, and asked “When you are hungry, which of the following sensations do you usually experience in the stomach?” The following sensations were evaluated: Emptiness; Rumbling; Hollowness; Tension and tightness; Aches and pains; Nausea; Relaxed; Fullness; and Distension and bloating. Block three concerned the mouth and participants were asked “When you are hungry, which of the following sensations do you usually experience in the mouth?” Each of the following sensations were evaluated: Salivation; Dryness; Emptiness; Tension and tightness; Unpleasant taste; Pleasant taste; and Relaxed. Block four asked about throat sensations, “When you are hungry, which of the following sensations do you usually experience in the throat?” Participants evaluated: Dryness; Emptiness; Tension and tightness; Unpleasant feeling; Pleasant feeling; Relaxed; and Nausea. Block five concerned sensations experienced in the head, “When you are hungry, which of the following sensations do you usually experience in the head?” This involved: Headache; Dizziness; and Fainting. Finally, block six concerned general bodily sensations, asking “When you are hungry, which of the following general physical sensations do you usually experience?” There were eight items to evaluate: Weakness; Tiredness and sleepiness; Lack of energy and fatigue; Restlessness; Trouble concentrating; Coldness; Warmth; and Energetic. In total, this part of the Hunger survey was composed of 43 questions (i.e., items). Details regarding its reliability are presented in Section 3 because of the centrality of this measure.

#### 2.2.2 Hunger beliefs questionnaire

As there is no measure available to assess participant beliefs about the two main models of hunger, we generated a range of statements pertinent to each, as well as drawing upon items from the only other study to examine hunger beliefs (Assanand, Pinel, & Lehman, 1998). All 43 statements were evaluated in the same way using a same 7‐point category scale (1 [*Strongly disagree*] to 4 [*Neither agree nor disagree*] to 7 [*Strongly agree*]). There were 23 items pertaining to homeostatic beliefs (e.g., I believe that when I am hungry I have low levels of blood sugar; A craving for a certain food means that I am missing certain nutrients from my body) and 20 concerning learning‐related hunger (e.g., The sight of food I like activates my hunger; I believe that sitting in a restaurant would increase my hunger). The 23 homeostatic items had a coefficient *α* = .78 and the learning items an *α* = .66, putting these in the acceptable range for a research‐related instrument (i.e., 0.5+; Nunnally, 1978).

#### 2.2.3 Three‐Factor Eating Questionnaire

This measure is widely used to assess eating attitudes that span from the normal into the pathological with the scale generating three dimensional scores—uncontrolled, restrained, and emotional eating. The revised 18‐item TFEQ (Cappelleri et al., 2009) was used, with this having good reliability for its three subscales (*α* = .78–.94, respectively).

### 2.3 Procedure

Both the student offspring and caregiver surveys were completed online using Qualtrics. All participants were asked to undertake the survey hungry and to do so around 30 min before they planned to eat a main meal.

Student offspring completed the surveys first. After consenting, they were asked to provide the name and contact details of the person who mainly looked after them as a child (primary caregiver). They then recorded their own age, height, weight, gender, whether they were currently dieting, the presence of any condition that might affect their capacity to experience hunger, and what they last ate. Participants then completed the Hunger survey and the Hunger beliefs questionnaire. Each of these was prefaced by two open questions to get participants thinking about the particular survey topic (Hunger survey: What does hunger feel like? How do you know if you are hungry? Hunger Beliefs: What causes hunger? What happens if hunger is ignored?). In addition, four check questions were also included within these surveys to aid identification of any respondent repeatedly clicking the same response button for every question. Two of these questions were placed into the main blocks of the Hunger survey (Loss of vision as a hunger sign; People need food to survive) and the other two were placed in the Hunger Beliefs questionnaire (Breakfast is normally eaten in the evening; Sugar has a sweet taste). These questions used the same response scales as the other items in their respective survey. Finally, the TFEQ was completed.

Once the student offspring had completed their surveys, the research team sent their primary caregiver an email invitation and the study link. Primary caregiver surveys were identical to the ones completed by their offspring, except that the caregiver had to provide the full name of their offspring (i.e., the person who asked them to take part), and their relationship to them. This was to ensure that offspring surveys could be matched to those of their caregiver.

### 2.4 Analysis

Data were suitable for parametric analysis as established by examination of skewness and kurtosis and use of the Shapiro–Wilks test. The exception was offspring BMI, which required transformation. For the regressions, requisite assumptions were met, with no multicollinearity and with normally distributed residuals. Alpha was set at .05 (two‐tailed) and adjusted, as reported in the text, for multiple comparisons using a Bonferroni correction.

## 3 RESULTS

### 3.1 Motivational state on completing the survey

For caregivers and offspring, the median time since they last ate was 2–4 h, with no significant difference in response between the two (Sign test, *Z* < 1). For hunger ratings, caregivers reported being “moderately hungry” (*M* = 2.8, *SD* = 0.9) as did their offspring (*M* = 2.9, *SD* = 0.8) and they did not differ in this regard (paired‐sample *t* = 1.18). For urge to eat ratings, caregivers reported a “moderate urge to eat” (*M* = 2.6, *SD* = 0.9), with this urge being somewhat stronger in their offspring (*M* = 2.9, *SD* = 0.8) (*t*(110) = 2.24, *p* = .027, *d* = 0.21, 95% confidence interval [CI]: 0.03–0.46). On the thoughts about food question, caregivers reported “a small to moderate” amount (*M* = 2.4, *SD* = 0.9), with this being similar in their offspring (*M* = 2.6, *SD* = 0.9) (*t* = 1.35). For how much food they could eat now, caregivers reported a “moderate amount of food” (*M* = 2.9, *SD* = 0.7) as did their offspring (*M* = 3.0, *SD* = 0.7; *t* = 1.88). In sum, both caregivers and offspring were similarly hungry, but the offspring reported a slightly greater urge to eat.

### 3.2 Caregiver–offspring similarity for the Hunger survey

Both the caregiver and offspring responses on the 43‐item Hunger survey had excellent internal reliability, with coefficient *α*s of .93 and .91, respectively.

For *each* caregiver–offspring pair, we computed a Pearson correlation between their responses on the 43‐item Hunger survey, yielding 111 correlations, with a mean caregiver–offspring *r* of .42 (*SD* = .23). These *r* values were then transformed into Fisher's *r*ʹ (for normalization) and compared to a *μ* of 0 using a one‐sample *t*‐test (i.e., to determine if they were significantly above zero). There was a significant difference (*t*(110) = 16.36, *p* < .001, Cohen's *d* = 1.55, 95% CI: 0.42–0.54), indicating that caregiver–offspring pair correlations were positive.

As responses on the hunger questionnaire between *any* two people might be somewhat similar, we undertook a further test. First, we randomly selected 500 pairs of responses between unrelated caregivers and offspring, and computed the correlation between their responses on the 43‐item Hunger survey (a value of 500 was used as it produced a stable *r*). These 500 random pair correlations had a mean *r* of .34 (*SD* = .22). Following Fisher transformation, this random‐pair *r*ʹ was used as the *μ* value in a further one‐sample *t*‐test on the actual caregiver–offspring pair correlation (*r*ʹ) data. There was a significant difference again (*t*(110) = 3.46, *p* < .001, Cohen's *d* = 0.33, 95% CI: 0.04–0.16), indicating that the actual caregiver–offspring pair correlations were still greater than that of unrelated caregiver and offspring pairs.

### 3.3 Caregiver–offspring factor scores on the Hunger survey and their relationship

The data from all 222 participants (i.e., all caregivers and offspring) were combined to determine the factor structure of the Hunger survey, so we could then establish the similarity between caregivers and offspring for each factor (we note that the outcomes are substantially similar if just the caregiver or offspring sample is used to determine the factor structure).

The combined sample data were factorable, with a Kaiser–Meyer–Olkin index of sampling adequacy of 0.89 (i.e., >0.5) and a significant Bartlett's test of sphericity (*χ* ^2^ [903] = 5035.6, *p* < .001). Factor analysis with Oblimin rotation was employed to allow some degree of correlation between factors. The communalities were quite high (*M* = 0.65), and as both the scree plot and Kaiser's criterion (i.e., eigenvalues > 1) indicated 10 factors, all were retained (see Table 1 for pattern matrix). Factor scores were then computed for each participant using the average intensity response for questions loading on that factor. Coefficient alpha was also calculated for each factor score (see Table 1), indicating good to very good reliability for all factors except Full stomach and Salivation.

**TABLE 1:** Pattern matrix of the Hunger survey data

Factor (% variance accounted for, coefficient alpha)										
	Factor number (only values > ± .3 are included)									
Items	1	2	3	4	5	6	7	8	9	10
1. Fatigue (27.3%, alpha = .81)										
Fatigue	.66					.37				
Weakness	.63						–.33			
Tiredness	.45									
Bad taste in mouth	.44									
Headache	.42									
2. Positive anticipation (12.9%, alpha = .81)										
Relaxed throat		.82								
Relaxed mouth		.74								
Pleasant feel throat		.55								
Pleasant taste mouth		.49								
Energetic		.46					–.45			
3. Oropharyngeal (4.6%, alpha = .88)										
Tension throat			–.85							
Bad feeling throat			–.74							
Emptiness throat			–.69							
Tension mouth			–.64							
Dry throat			–.56							
Empty mouth			–.49						.31	
4. Nausea (4.2%, alpha = .84)										
Stomach nausea				–.88						
Throat nausea				–.75						
Stomach ache				–.64						
Feel faint	.42			–.59						
Feel dizzy	.47			–.47						
Stomach tension			–.37	–.40						
5. Positive mood (3.2%, alpha = .84)										
Cheerful					–.82					
Calm					–.81					
Excited					–.74					
Content					–.72					
Relaxed stomach	.42				–.46					
6. Full stomach (3.1%, alpha = .50)										
Bloated						.70				
Full						.66				
Cold						.33				
7. Tension (2.7%, alpha = .70)										
Apprehensive							–.69			
Nervous							–.42			
Poor concentration							–.37			
Warm							–.34			
8. Salivation (2.6%, alpha = –.16)										
Mouth salivation								–.77		
Mouth dry			–.48					.49		
9. Empty stomach (2.5%, alpha = .71)										
Stomach hollow									.78	
Stomach empty									.76	
Stomach rumbling									.46	
10. Irritable (2.4%, alpha = .72)										
Bored										.89
Impatient										.40
Restless										.37
Irritable										.35

Mean values for each of the factor‐derived scores for caregivers and their offspring are presented in Table 2. The 10 factor scores differed in intensity from “Very weak” (a score of 2) to “Strong” (a score of 5), with most being around a score of 3, equivalent to a response of “Weak.” The ordering of the factor scores in terms of intensity was the same for caregivers and offspring, with Fatigue and an Empty stomach being the most intensely reported internal hunger signals. However, offspring consistently reported experiencing these internal hunger signals more intensely than their caregivers. This difference was significant for seven out of the 10 factors, with a small‐to‐medium effect size.

**TABLE 2:** Pearson correlations between caregiver and offspring factor scores and tests of difference in mean intensity for each factor

	Caregiver	Offspring	Comparison	Correlation
Factor name	*M* (*SD*)	*M* (*SD*)	*t*_110_ = (*d*), 95% CI	*r*_109_ = (*d*)
1. Fatigue	3.4 (1.2)	4.0 (0.9)	5.06* (0.48), 0.35–0.80	.36* (0.77)
2. Positive anticipation	1.9 (0.9)	2.0 (0.8)	1.50 (0.14), −0.05 to 0.36	.21 (0.43)
3. Oropharyngeal	2.6 (1.3)	3.2 (1.1)	5.28* (0.50), 0.40–0.87	.46* (1.04)
4. Nausea	2.5 (1.1)	3.2 (1.1)	5.90* (0.56), 0.49–0.99	.28* (0.58)
5. Positive mood	2.1 (0.9)	2.3 (0.8)	1.92 (0.18), 0.00–0.44	.11 (0.22)
6. Full stomach	1.9 (0.9)	2.2 (0.9)	2.96* (0.28), 0.09–0.45	.44* (0.98)
7. Tension	2.7 (1.0)	3.2 (0.8)	4.14* (0.39), 0.23–0.65	.26 (0.54)
8. Salivation	3.1 (1.1)	3.5 (0.9)	2.54 (0.24), 0.07–0.59	.07 (0.14)
9. Empty stomach	4.3 (1.1)	4.8 (0.8)	4.22* (0.40), 0.25–0.68	.27* (0.56)
10. Irritable	3.4 (1.1)	4.2 (0.9)	6.60* (0.63), 0.56–1.05	.15 (0.30)

Abbreviation: CI, confidence interval.

*Significant (Bonferroni adjusted, *p* < 0.005).

Pearson correlations were then calculated for each factor using the item averaged scores, between caregivers and their offspring. Alpha was set at .005 using a Bonferroni correction. All 10 of the caregiver–offspring correlations were positive, and the results are detailed in Table 2. Significant relationships between caregivers and their offspring were evident for five of the factors—all with medium to large effect sizes. These caregiver–offspring similarities covered both diffuse (Fatigue, Nausea) and focal sensations (Oropharyngeal, Full stomach, Empty stomach). No relationships were evident for irritability, tension (this approached significance, *p* = .0058), salivation, or the two affectively positive factors (positive anticipation, positive mood).

### 3.4 Potential moderators of caregiver–offspring similarity on the Hunger survey

Seven potential moderating variables were examined: TFEQ scales (uncontrolled, restraint, emotional), BMI, gender, and participants belief strength concerning homeostatic and learning‐based models of hunger. Details about these variables are presented in Table 3.

**TABLE 3:** Potential moderating variables and correlations between caregiver and offspring

	Caregiver	Offspring	Comparison	Correlation
Variable	*M*/*n* (*SD*)	*M*/*n* (*SD*)	*t*_110_ = (*d*), 95% CI	*r*_109_ = (*d*)
TFEQ uncontrolled	2.1 (0.6)	2.5 (0.5)	4.68* (0.44), −0.40 to 0.19	.10 (0.20)
TFEQ restraint	2.5 (0.7)	2.3 (0.7)	2.37* (0.23), 0.04–0.42	.01 (0.02)
TFEQ emotional	2.1 (0.7)	2.4 (0.8)	2.64* (0.25), −0.40 to 0.06	.06 (0.12)
Gender	6 male	17 male	–	–
BMI^a^	26.3 (5.1)	24.2 (6.2)	*Z* = 4.49^a^*	.33^a^* (0.70)
Homeostatic beliefs	4.6 (0.7)	4.8 (0.5)	1.50 (0.14), −0.25 to 0.04	.25* (0.52)
Learning beliefs	4.6 (0.6)	4.7 (0.5)	1.46 (0.14), −0.24 to 0.04	.11 (0.22)

Abbreviation: CI, confidence interval.

^a^ Tested nonparametrically using a Wilcoxon test and Spearman's rho.

*p < .05.

For the TFEQ scales, offspring uncontrolled and emotional eating were higher on average than for their caregivers, with this reversed for restraint. There was no relationship between offspring and caregivers TFEQ dimensions. For gender, both samples were mainly female. Caregivers had a significantly higher BMI than their offspring, and there was a significant correlation between caregiver and offspring BMI. For the belief scores, the means equate to a response of “Somewhat agree,” reflecting positive agreement with both homeostatic and learning‐based models of hunger (one‐sample *t*‐test indicate that belief scores in Table 3 all significantly exceed a response of “Neither agree nor disagree” [i.e., *μ* = 4]; all *t*s > 9.83). There was no significant difference in hunger beliefs between caregivers and offspring. While beliefs about learning‐based models of hunger were not related between caregivers and offspring, there was a significant positive relationship for homeostatic beliefs.

The 111 Fisher‐transformed correlations between caregivers and offspring on the 43‐item Hunger survey served as the dependent variable for the initial analysis (see Section 3.2). We conducted a regression with simultaneous entry of both caregiver and offspring BMI, gender, TFEQ dimensions (uncontrolled eating, restraint, emotional eating), and belief scores (homeostatic, learning) as potential predictors of the strength of the caregiver–offspring Hunger survey relationship.

**FIGURE 1:**
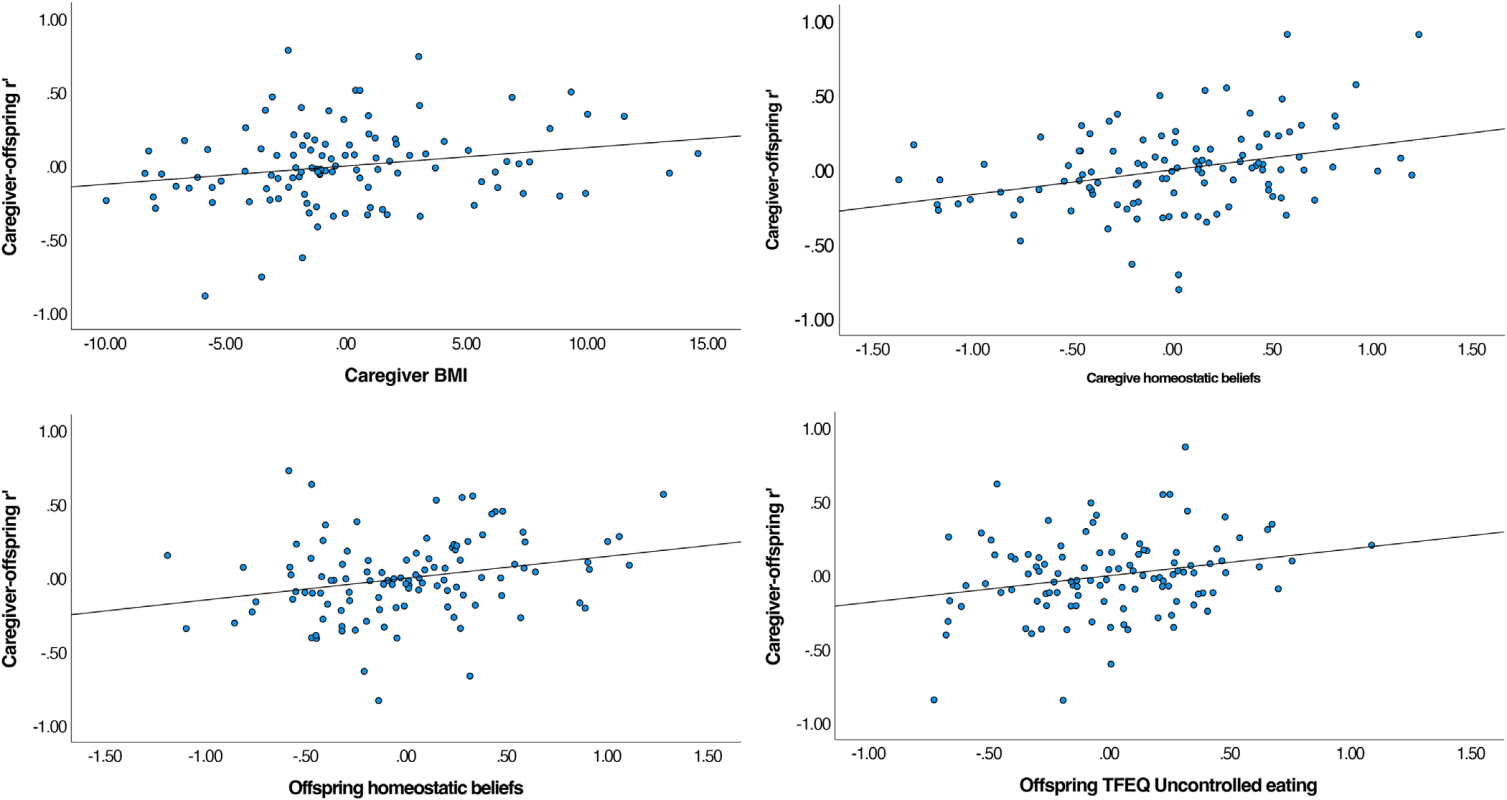
Standardized residual plots illustrating the relationship between caregiver BMI, caregiver homeostatic beliefs, offspring homeostatic beliefs, and offspring uncontrolled eating with caregiver–offspring correlation on the 43‐item Hunger survey.

Data from this analysis are presented in Table 4. The overall model was significant (*F*(14,95) = 2.75, *p* = .002, root mean square error = .08, adjusted *R* ^2^ = .18). There were four variables that each made a significantly unique contribution to the model: caregiver homeostatic beliefs, offspring homeostatic beliefs, offspring TFEQ uncontrolled eating, and caregiver BMI. Greater caregiver and offspring belief in a homeostatic model of hunger, greater offspring TFEQ uncontrolled eating, and greater caregiver BMI were all associated with greater caregiver–offspring similarity on the 43‐item hunger questionnaire (see Figure 1).

**TABLE 4:** Regression analysis with caregiver (C) offspring (O) Hunger survey correlations as the dependent variable (DV)

			Correlation with DV		
Predictor	*B* (*SE*), 95% CI	Beta	*r*_0_	*Squared semi-partial correlation coefficient* ^2^	*p*
C TFEQ uncontrolled	–0.02 (0.07), −0.16 to 0.11	–0.04	.14	.00	.74
C TFEQ restraint	0.00 (0.04), −0.07 to 0.08	0.01	.08	.00	.97
C TFEQ emotional	0.07 (0.05), −0.03 to 0.17	0.16	.10	.01	.19
C gender	–0.06 (0.13), −0.31 to 0.20	–0.04	–.04	.00	.64
C BMI	0.01 (0.01), 0.00–0.02	0.21	.13	.04	.029
C homeostatic beliefs	0.17 (0.05), 0.07–0.26	0.37	.34	.09	.001
C learning beliefs	–0.07 (0.06), −0.19 to 0.05	–0.14	.13	.01	.24
O TFEQ uncontrolled	0.18 (0.08), 0.03–0.33	0.30	.24	.04	.018
O TFEQ restraint	–0.01 (0.04), −0.10 to 0.08	–0.03	.06	.00	.77
O TFEQ emotional	–0.01 (0.05), −0.10 to 0.09	–0.01	.14	.00	.91
O gender	0.03 (0.08), −0.13 to 0.19	0.04	.03	.00	.71
O BMI	–0.19 (0.24), −0.67 to 0.28	–0.08	–.07	.00	.42
O homeostatic beliefs	0.15 (0.06), 0.03–0.26	0.26	.33	.05	.011
O learning beliefs	–0.06 (0.08), −0.23 to 0.10	–0.09	.16	.00	.46

Abbreviation: CI, confidence interval.

For the caregiver and offspring factor score data from the Hunger survey, a different approach was adopted. Each caregiver factor score was subtracted from its associated offspring factor score, yielding 10 factor difference scores (i.e., an indication of caregiver–offspring dissimilarity for each factor). Each of these 10 factor difference scores then served as the dependent variable in a series of exploratory regression analyses (hence no adjustment of alpha), with each using just the four predictors identified in the analysis above (i.e., caregiver BMI, caregiver homeostatic beliefs, offspring TFEQ uncontrolled eating, and offspring homeostatic beliefs).

The 10 regression analyses are summarized in Table 5. Two things are noteworthy. First, offspring homeostatic beliefs are unique predictors of offspring–caregiver response dissimilarity in five cases. For each, greater offspring belief in a homeostatic model of hunger is linked to a more intense hunger response on that factor, relative to their caregiver. Second, caregiver homeostatic beliefs are unique predictors of offspring–caregiver response dissimilarity in eight cases, with a positive association in two and a negative association in six. For the positive associations, greater caregiver belief in a homeostatic model of hunger was linked to a more intense hunger response on those factors in the offspring, relative to their caregiver. For the negative associations, greater caregiver belief in a homeostatic model was linked to offspring having a less intense hunger response on those factors, relative to their caregiver.

**TABLE 5:** Regression summary for caregiver–offspring Hunger survey factor scores

Dependent variable	Model	Significant unique predictors (*Sr*)	
1. Fatigue	*F*(4, 105) = 5.52, *p* = .001, *R^2^* = .14	OS HB (.27)	CG HB (–.37)
2. Positive anticipation	*F*(4, 105) = 2.63, *p* = .038, *R^2^* = .06		CG HB (.26)
3. Oropharyngeal	*F*(4, 105) = 2.45, *p* = .051, *R^2^* = .05	OS HB (.23)	
4. Nausea	*F*(4, 105) = 4.49, *p* = .002, *R^2^* = .11	OS HB (.28)	CG HB (–.31)
5. Positive mood	*F*(4, 105) = 1.66, *p* = .165, *R^2^* = .02		CG HB (.23)
6. Full stomach	*F*(4, 105) = 0.14, *p* = .967, *R^2^* = .00		
7. Tension	*F*(4, 105) = 2.97, *p* = .023, *R^2^* = .07	OS HB (.19)	CG HB (–.29)
8. Salivation	*F*(4, 105) = 3.82, *p* = .006, *R^2^* = .09	OS HB (.26)	CG HB (–.26)
9. Empty stomach	*F*(4, 105) = 1.70, *p* = .156, *R^2^* = .03		CG HB (–.23)
10. Irritable	*F*(4, 105) = 1.74, *p* = .148, *R^2^* = .03		CG HB (–.22)

Abbreviations: CG HB, caregiver homeostatic beliefs; OS HB, offspring homeostatic beliefs.

## 4 DISCUSSION

We tested here for similarity between caregiver and offspring internal signals of hunger. Our data indicate, overall, substantial similarity between offspring and caregivers in this regard. The hunger survey data were factorable, revealing 10 factors. Using a conservative alpha (.005), five factors were significantly correlated between caregiver and offspring, with all 10 being positive. We also examined variables that might moderate the similarity of offspring–caregiver responses on the Hunger survey. Greater similarity between offspring and caregiver responses was evident when (1) caregivers or their offspring held strong beliefs in homeostatic models of hunger; (2) offspring scored high on uncontrolled eating; and (3) caregivers had a higher BMI. Finally, we examined whether these moderating variables were related to differences in the factor scores between offspring and caregiver. In the four significant models, the pattern was similar. Caregivers who held strong beliefs in a homeostatic model of hunger were more likely to have offspring with similar hunger intensity scores for that factor. Offspring who had high levels of belief in homeostatic models of hunger tended to have higher hunger intensity scores for that factor, relative to their caregivers. Indeed, belief in homeostatic models of hunger—which are not well supported scientifically as we outlined in Section 1 (Rogers & Brunstrom, 2016; Stricker, 1984; Strubbe & Woods, 2004; Woods et al., 2000)—seems to be quite a powerful moderator of caregiver–offspring similarity in internal hunger states.

There are two caveats that need to be kept in mind when considering these findings. First, twin studies indicate that many aspects of ingestive behavior are heritable. This is the case for BMI (Wallis & Raffan, 2020), the three dimensions of the TFEQ (Steinle et al., 2002), and reports of hunger (De Castro, 1999; Stevenson et al., 2015). This raises the possibility that the caregiver–offspring similarities reported here might arise from shared genetic heritage. While we would contend that learning is a key factor, not least because holding beliefs about an erroneous model of hunger seem to be important for moderating offspring–caregiver similarity (discussed further below), the possibility remains that genetic dispositions could favor learning one form of internal signal over another. Twin studies offer one route for addressing this. Another is to see if parents actually “teach” their children what hunger is.

A second caveat concerns gender. Most caregivers were mothers. This is not surprising, and especially so for studies examining feeding, which often falls to the female caregiver (e.g., Dupuy et al., 2021; Galloway et al., 2010). The student–offspring sample was also predominantly female. While we did not detect any moderating influence of gender in the regression analysis, a more robust answer requires a larger number of male offspring, and male caregivers. We note that in Monello and Mayer's (1967) study, there were some gender differences in reported outcomes on the Hunger survey. Males had a more restricted range of internal cues, and females tended to have lower hunger intensity scores. But in the main, their responses were similar and so we *suspect* that any differences between genders may be more in how offspring–caregiver similarity is moderated, rather than in whether or not it is present.

There are two reasons for thinking that learning is important in understanding caregiver–offspring similarity here. The first is theoretical and concerns meaning, namely that an internal sensation cannot have meaning until this is discovered by interaction with the environment (e.g., Harshaw, 2008). This makes it highly likely that some form of learning process occurs—as suggested by Hebb (1949), Bruch (1969), and Changizi et al. (2002). The second concerns the finding that caregiver and offspring beliefs influence caregiver–offspring similarity. This would seem to suggest the operation of psychological processes shaping which internal state becomes linked to hunger, and perhaps also the degree of urgency and meaning attached to that internal state. As homeostatic models are premised around the notion of fuel depletion (Assanand, Pinel, & Lehman, 1998; Rogers & Brunstrom, 2016), the appealing analogy is that of the petrol tank emptying. This does imply a certain urgency in noticing this state and acting upon it. It may be then that caregivers who hold homeostatic beliefs exert a stronger influence over their offspring's hunger (to avoid “running out of fuel”), so producing greater offspring–caregiver similarity. And where the offspring hold strong homeostatic beliefs themselves, this may lend their own internal hunger cues a certain urgency, above and beyond those of their caregivers.

If some form of learning process is likely, it is important to consider what form it might take. As there appears to have been no study of this, we suggest that when a child displays some particular behavior (e.g., tiredness, rumbling stomach, irritability, etc.) or reports some particular state that is consistent with their caregiver's understanding of hunger, the meaning of this state is communicated to the child (e.g., “you're hungry!”). In addition, as the caregiver is also likely to believe that this signals the need for food, then the observed child “hunger cue” will often be followed by food. If that food is then enjoyed, that internal state can then serve as an occasion setter in the manner described in Section 1 (Changizi et al., 2002; Davidson, 1993; Holland, 1991). Of the five factors where there was significant caregiver–offspring similarity, Fatigue, Oropharyngeal, Nausea, Full stomach, and Empty stomach, all of these could conceivably be identified, and linked to feeding as occasion setters. Indeed, an argument could be made that these five represent the more readily associable internal states. This is something that really needs data, with attempts needed to catch such learning in progress, plus studies of caregiver beliefs about these internal states while they are raising their child.

There has been considerable interest in whether certain aspects of caregiver food‐related behavior have negative impacts on the capacity of the child to adequately regulate their food intake and ultimately their weight (Beckers et al., 2021; Farrow & Blissett, 2008; Galloway et al., 2010). Three key factors have been identified—pressure to eat, palatable food restriction, and using food as a reward. The concern is that these train children to attend to the wrong cues—namely external indicators of when to start and stop eating. This focus shares much in common with the intuitive eating movement, and the idea that careful attention to internal states offers an “honest” guide on when to start and stop eating (e.g., Tribole & Resch, 1995). At least in the case of starting eating, internal states of hunger are believed to be important precisely because they are thought to reflect fuel need. The findings from this study suggest that internal cues that signal hunger may not be especially privileged. That is, they reflect learning, or at least a learning component, and simply signal when food is likely to taste good.

In conclusion, we find strong evidence of offspring–caregiver similarity in internal signals of hunger. This similarity is moderated by beliefs that caregivers and their offspring have over the causes of hunger, with erroneous homeostatic beliefs being especially potent.

## CONFLICT OF INTEREST STATEMENT

The authors declare no conflicts of interest.

## Data Availability

Data are available from the first author on request.
